# Certification Criteria and Bio-Inspired Functional Themes: LEED v5 and BREEAM V7 Profiles with a Bounded YeS-TR Audit

**DOI:** 10.3390/biomimetics11100737

**Published:** 2026-10-14

**Authors:** Ezgi Yılmaz, Mehmet Sair Akkam

**Affiliations:** 1 Department of Architecture, Faculty of Architecture and Design, Istanbul Aydın University, Istanbul 34295, Türkiye; 2 Faculty of Engineering and Architecture, İstanbul Gelişim University, Istanbul 34310, Türkiye; msakkam@gelisim.edu.tr

**Keywords:** biomimetics, bio-inspired design, green building certification, sustainability assessment, LEED v5, BREEAM V7, YeS-TR, Türkiye

## Abstract

This study examines whether green building rating system architectures formally recognize functions associated with bio-inspired design. It compares the complete LEED v5 BD+C New Construction dataset (49 criteria; 110 points) and office-applicable BREEAM International New Construction V7 (51 issues; 165 standard credits). A Biomimetic Readiness Lens (BRL) provisionally regroups the eleven-domain Harmonized Sustainability Taxonomy into six interpretive dimensions. The mapping is deterministic and exploratory; it adds an interpretive synthesis beyond the underlying sustainability-domain results but is not independently validated and does not measure biological emulation, building performance, or design outcomes. Coverage, scoring incentives, minimum requirements, and qualitative localization are analyzed separately. Results show that resource-related criteria are prominent in both complete systems, while their credit allocation and minimum-standard structures differ. Independent recoding of all 100 items found complete agreement on primary HST domains and limited agreement on optional secondary codes; all 54 secondary differences were subsequently adjudicated under a source-based coding rule. The reconciled classifications retain 34 optional secondary assignments. The initial reliability estimates are reported separately from post-recoding adjudication. Document comparability and national source coverage also constrain interpretation. The BRL should be read as a provisional analytical crosswalk, not as a validated measure of biomimetic readiness.

## 1. Introduction

Biomimetics translates principles, functions, and mechanisms observed in living systems into human design and technological applications. In architecture, this transfer extends beyond formal imitation to functional strategies such as circular resource use, efficient energy and material flows, environmental responsiveness, resilience, distributed networks, self-regulation, and cooperation [1,2,3]. Recent construction research further indicates that biomimicry adoption is shaped not only by design knowledge and technology but also by policy, institutional support, education, and regulatory frameworks [4]. This suggests that the enabling environment around design deserves explicit biomimetic analysis alongside the building component, material, or envelope itself.

The present study shifts attention from biomimetic objects to the assessment infrastructure that shapes building decisions. Green building rating systems (GBRSs) are not treated as biomimetic systems, and certification compliance is not considered evidence of biological emulation. We ask whether formal criteria and governance mechanisms recognize selected functions that the biomimetic design literature associates with resource cycling, adaptation, ecological fit, networks, and coordination. This is an institutional-level exploratory analysis; it does not measure biomimetic design intent, building performance, or whether certification enables or constrains a design outcome.

GBRSs have become influential instruments for translating broad sustainability objectives into design requirements, verification procedures, and certification outcomes. Their global proliferation has supported a more structured treatment of energy, water, materials, indoor environmental quality, land use, and management within building projects, while also creating a heterogeneous landscape of criteria, weights, prerequisites, and verification mechanisms [5,6]. Because these systems were developed within different institutional, climatic, regulatory, and market contexts, similar sustainability objectives are not necessarily expressed or prioritized in the same way across rating tools [7,8].

Comparative GBRS research has therefore increasingly moved beyond simple category-by-category description. Earlier work demonstrated that widely used systems share substantial environmental concerns but may differ in the number of issues assessed, scoring thresholds, and the relationship between mandatory and elective requirements [9]. Methodological studies have likewise shown that normalization into common macro-areas is necessary when systems use different category structures and weighting logics [8]. More recent work in *Sustainable Cities and Society* combined rating-system evolution, criteria comparison, and certified-project data to evaluate the development of Chinese and U.S. green building standards, illustrating the value of linking system architecture with national policy trajectories rather than treating certification manuals as static checklists [10]. Direct precedents include content analysis of biomimicry principles in Malaysia’s Green Building Index, examination of biomimicry within BREEAM’s energy category, and work on integrating biomimicry design thinking with LEED [11,12,13]. The present study differs by comparing the current certification architecture through a transparent sustainability taxonomy and by stating the limits of an interpretive crosswalk derived from the Harmonized Sustainability Taxonomy (HST).

A second line of research has emphasized localization. International rating systems can provide mature and transferable assessment structures, but their effectiveness depends on how well they respond to local environmental pressures, construction practices, codes, resource constraints, and policy goals. Awadh [7] argued that regional agendas and local context should be central to rating-system design, while comparative research has shown that national and international systems may pursue similar sustainability aims through different regulatory and technical pathways. This issue has become particularly important as countries develop domestic rating frameworks that incorporate national standards and government implementation mechanisms rather than relying exclusively on imported voluntary systems.

The decarbonization agenda has further changed the expectations placed on GBRSs. Olanrewaju et al. [14] found that operational credits substantially outnumber embodied-carbon credits across multiple certification systems, while Ng et al. [15] similarly concluded that most GBRSs continue to prioritize operational carbon and differ in life-cycle boundaries, verification mechanisms, and governance maturity. For Türkiye specifically, Arslan and Ulubeyli [16] compared YeS-TR, LEED, BREEAM, and DGNB through a whole-life-carbon framework and reported particularly strong carbon alignment for YeS-TR and LEED. These studies establish carbon alignment as an important comparison axis, but they also leave a different question unresolved: how sustainability priorities are institutionally expressed through the combined architecture of criterion coverage, scoring incentives, prerequisites, social/governance requirements, and national regulatory localization.

Recent methodological work reinforces the need to treat harmonization and governance explicitly. Kaufmann and Zhu [17] show that environmental, social, and governance indicators are distributed unevenly across building-level assessment tools and require a transparent harmonization framework for meaningful comparison. De Garrido-Talavera [18] likewise proposes a taxonomic structure intended to make GBRS adaptable across environmental and socioeconomic contexts. At the system-evolution level, Dharmarathna et al. [19] demonstrate that changes in rating tools, certification rules, and policy context can materially reshape certification practice over time. The emerging 2026 YeS-TR literature has begun to examine three complementary questions: building-level credit attainment in an existing residential case [20], alignment of YeS-TR criteria with the Sustainable Development Goals [21], and the prospective applicability of YeS-INT alongside international certification systems in developing-country contexts [22]. These studies confirm that the Turkish certification literature is moving from system emergence toward application and internationalization. However, they do not resolve how certification priority should be measured when coverage, point incentives, mandatory minima, multidomain functions, and regulatory localization operate simultaneously.

Türkiye provides a national context for the bounded audit. The 2020 study by Yılmaz and Balyemez [23] documented international certification use and developing domestic initiatives. The amendment published on 11 March 2025 (Official Gazette No. 32838) requires certification from 1 January 2026 for newly permitted public health, education, dormitory and service buildings with a total construction area of at least 10,000 m^2^ [24]. This policy context is not evidence of biomimetic performance.

Despite this transition, five analytical problems remain. First, direct comparisons can mislead when criterion counts, point weights, credits, and mandatory requirements are treated as equivalent measures. Second, single-domain coding can conceal cross-cutting sustainability objectives. Third, localization is often discussed descriptively rather than separated from the scoring structure. Fourth, source completeness varies across current systems. Fifth, comparisons rarely make explicit whether certification structures recognize functions discussed in bio-inspired design research. This study addresses these problems through source-controlled coding and an explicitly asymmetric design: complete system profiles are reported only for LEED and BREEAM, while YeS-TR is limited to a purposive criterion audit and documentary governance timeline.

The principal contribution is a provisional Biomimetic Readiness Lens (BRL) that groups HST findings into resource, ecological, adaptive, network, coordination, and social themes. This regrouping makes the concentration and limited representation of selected bio-inspired functional themes visible within certification architecture. The contribution is organizational and interpretive: the BRL adds no new observations and does not establish biological function, biomimetic intent, or predictive validity. Separate coverage, scoring, and requirement diagnostics and four alternative allocations of secondary codes support this interpretation. A bounded YeS-TR audit and documentary timeline provide the national institutional context.

## 2. Materials and Methods

### 2.1. Research Design

This study uses comparative structured content analysis and documentary timeline analysis to examine green building certification in Türkiye [25]. It treats certification as an architecture of environmental assessment, professional governance, regulatory localization, and sustainability policy. The documentary timeline is not a repeated-measures or longitudinal performance study.

The study has two analytical levels. The first examines two complete international benchmarks: LEED v5 Building Design and Construction (BD+C) New Construction, coded at the criterion level, and BREEAM International New Construction V7, coded at the office-applicable issue level. The second examines selected current YeS-TR criteria through an audited matched-domain comparison and reviews documentary milestones in national certification governance.

The design reflects differences in source completeness and native scoring architecture. LEED v5 BD+C New Construction was coded from the official February 2026 Rating System; BREEAM V7 was coded from its Technical Manual. All 49 LEED records were subsequently checked against the July scorecard and the 31 July 2026 addenda [26,27]. New Construction record membership, prerequisite/credit status, and maximum points were unchanged, while requirement and guidance revisions were identified. The 49-record edition audit is provided in Supplementary Data S1. The published HST classifications remain a February-edition coding snapshot; the edition check is not a full independent recoding of July requirements. For YeS-TR, the amended regulation and 2026 public-building implementation guidance were consulted, but the official annex was not extracted criterion by criterion. The guide supports wording/status verification only for the 15 audited criteria; their official annex point values were not verified. No system-wide YeS-TR weighting profile is calculated [28,29,30,31].

The asymmetric evidence-depth design avoids presenting incomplete evidence as a complete system profile. LEED and BREEAM are treated as complete benchmarks but analyzed using their native scoring structures. B.E.S.T.-Ticari is mentioned only as contextual background because a complete current criterion-level primary manual was not available for reproducible extraction; it is not part of the analysis [31,32].

The 2020 study by Yılmaz and Balyemez [23] serves only as a historical documentary reference point. No scores or criterion classifications from that study were transferred to the current dataset.

### 2.2. Research Questions and Analytical Framework

The study addressed six research questions:

- RQ1. How does the provisional Biomimetic Readiness Lens regroup observed sustainability coverage into six bio-inspired functional themes, and what limits its interpretation?
- RQ2. How do LEED v5 and BREEAM International New Construction V7 structure sustainability priorities through criterion/issue coverage, scoring incentives, and mandatory or minimum-standard mechanisms?
- RQ3. How are management, social equity, indoor environmental quality, energy, water, and site/mobility priorities operationalized in the audited YeS-TR criteria, and what conceptual correspondences can be identified with selected LEED v5 criteria?
- RQ4. How robust are LEED v5 and BREEAM V7 domain priorities to alternative allocations of explicitly coded secondary sustainability functions?
- RQ5. Which selected documentary milestones changed the formal institutional position of green building certification in Türkiye between the 2020 reference context and 2026?
- RQ6. Which national professional, verification and regulatory mechanisms are illustrated by the purposive YeS-TR criterion audit?

The framework combines full criterion-level coding of LEED v5, office-applicable issue-level coding of BREEAM V7, the 11-domain HST, a 15-criterion YeS-TR audit, and alternative-allocation sensitivity analysis. A documentary timeline summarizes selected governance milestones. The BRL is a secondary interpretation of the HST profiles, not a new criterion-level biological coding scheme.

### 2.3. Selection and Scope of Certification Systems

LEED v5 BD+C New Construction was selected as the principal international benchmark because an official primary rating-system document was available and because the scheme provides an explicit structure of prerequisites, credits, and point allocations suitable for quantitative content analysis. The analysis was restricted to the New Construction pathway to maintain consistency with the building-level scope of this study. Interior-only, neighborhood-scale, operations-only, and existing-building-only pathways were not incorporated into the quantitative dataset [30]. The coded LEED profile uses the February 2026 base edition. The July scorecard and addenda were screened for all 49 records [26,27]; changes are documented in Supplementary Data S1. The results retain February-edition HST coding rather than claiming a fully recoded July profile.

A total of 49 LEED v5 New Construction prerequisites and credits were extracted and coded. The dataset recorded the original category, criterion code and name, prerequisite or credit status, maximum available points, primary and secondary sustainability domains, criterion subdomain, performance-based status, quantitative-threshold status, verification mechanism, lifecycle stage, regional adjustment, explicit climate content, explicit carbon content, explicit resilience content, source location, and coding rationale [30].

BREEAM International New Construction Version 7.0.0 (SD6073; issue date 16 July 2025) was analyzed using the non-residential fully fitted pathway. Residential-only variants—Hea 09 Private space, Tra 02b Sustainable transport measures, and Wst 03b Operational waste—were excluded, yielding 51 office-applicable assessment issues and 165 standard credits. Category weights were not converted into a Türkiye-specific profile because Version 7 adapts weights to the country or regional context. Raw standard-credit shares are not directly equivalent to LEED normalized point shares. Rating benchmarks are Pass (≥25%), Good (≥40%), Very Good (≥55%), Excellent (≥70%), and Outstanding (≥85%); achievement also depends on rating-specific minimum standards [31].

YeS-TR was selected as Türkiye’s national certification framework. We purposively retained 15 criteria from Bütünleşik Bina Tasarım, Yapım ve Yönetimi (BBT), İç Ortam Kalitesi (İOK), Enerji Kullanımı ve Verimliliği (EKV), and Su ve Atık Yönetimi (SAY). Inclusion required a verifiable current wording or status in the post-amendment official framework or 2026 guidance; applicability to the office/non-residential or public-building pathway; sufficiently explicit content for HST coding; and a defensible conceptual comparator. The four categories were selected to cover technical and process functions available in the verified sources; the set is not a maximum-variation sample and includes transport connections within the bundled site criterion BBT 01 K4, rather than a dedicated mobility criterion. The full candidate count and exclusion-by-criterion log were not retained, so selection reproducibility is limited. Findings are descriptive and are not a YeS-TR system profile [28,29].

### 2.4. Harmonized Sustainability Taxonomy

Because certification systems organize sustainability requirements using different terminology, category boundaries, scoring structures, and prerequisite mechanisms, a Harmonized Sustainability Taxonomy (HST) was developed as a common analytical structure independent of original category names. This approach follows the broader logic of harmonization in comparative GBRS research while extending it to climate resilience, contextual priorities, and social/governance dimensions that have gained prominence in recent sustainability-assessment work [8,17,18]. The final taxonomy contained 11 domains:

- HST-1—Energy and Operational Carbon;
- HST-2—Embodied Carbon, Materials and Circularity;
- HST-3—Water;
- HST-4—Site, Land and Ecology;
- HST-5—Indoor Environmental Quality and Health;
- HST-6—Transport and Mobility;
- HST-7—Pollution and Environmental Externalities;
- HST-8—Management and Performance Verification;
- HST-9—Climate Resilience and Adaptation;
- HST-10—Innovation, Regional and Contextual Priorities;
- HST-11—Social Equity, Accessibility and Community Well-being.

HST-11 was introduced during pilot coding because several current requirements could not be validly classified under environmental or indoor-quality categories alone. These included human-impact assessment, equitable development, accessibility, community-oriented space, and worker health and safety [29,30].

### 2.5. Biomimetic Readiness Lens

The Biomimetic Readiness Lens (BRL) is a provisional interpretive crosswalk between selected HST domains and functional themes discussed in the biomimicry and bio-inspired design literature [1,2,3,4]. Its construct validity has not been established through expert elicitation, biological-model tracing, known-groups comparison, or application to explicitly biomimetic buildings. It is therefore not a validated measure of biomimetic readiness. Because it is derived deterministically from HST, it measures how conventional sustainability coverage can be reorganized under those themes; it cannot distinguish biomimetic intent from sustainability coverage or detect functions absent from the HST.

The mapping is deliberately narrow; Table 1 summarizes the operational dimensions, conceptual principles, and HST mapping. HST-1, HST-2, and HST-3 map to resource frugality and circular flows; HST-4 and HST-7 to ecological integration and externalities; HST-9 alone to adaptive responsiveness and resilience; HST-6 to network and mobility; HST-8 to coordination and verification; and HST-11 to cooperation, accessibility, and collective well-being. HST-5 (indoor environmental quality) is excluded from adaptive responsiveness because static comfort, air-quality, illuminance, or exposure thresholds do not themselves demonstrate adaptation. HST-10 (innovation, regional, and contextual priorities) is also left unmapped: contextual relevance does not necessarily establish ecological integration. HST-8 is not interpreted as biological feedback unless a criterion specifies a monitoring-and-response loop; the aggregate HST mapping indicates coordination/verification only. Life-friendly chemistry, self-assembly, form-based passive adaptation, and ecological mutualism as distinct from site protection are not represented reliably by the current HST.

For complete LEED and BREEAM profiles, the BRL aggregates primary HST coverage within each mapped dimension; the denominator remains the complete criterion or issue set. YeS-TR is excluded from BRL system-profile tables because its 15 purposively selected criteria are not representative. The BRL adds interpretive grouping, not new coded information. Secondary-domain incidence is kept separate and is not treated as a measure of biomimetic multifunctionality. Each record allows only one optional secondary domain. This field therefore cannot represent the number of additional functions, their relative strength, or their integration into a biological mechanism.

### 2.6. Criterion Coding and Quantitative Indicators

The individual prerequisite, credit, or mandatory requirement was the unit of analysis. Each item received one primary HST code based on its dominant explicit normative objective. A secondary code was added only when the source specified a distinct additional sustainability outcome or process that had its own requirement, threshold, or verification route. Generic co-benefits, broad associations, administrative roles, and local equivalency alone did not qualify. This threshold is an author-defined coding rule, not a validated scale.

Mandatory requirements and point-bearing credits were treated as analytically distinct structures. A prerequisite or mandatory requirement was therefore not interpreted as a zero-point criterion. This distinction prevented systems that operationalize sustainability through mandatory minima from appearing to assign no importance to those topics simply because no optional points were available.

For LEED, the Domain Coverage Index, Normalized Weight Share, Mandatory Requirement Density, and coverage-weight gap are defined as follows:

DCI_d = (n_d/N) × 100(1)

NWS_d = (P_d/ΣP) × 100(2)

MRD_d = (M_d/M) × 100(3)

ΔCW_d = NWS_d − DCI_d(4)

For BREEAM, Domain Coverage Index and Raw Credit Share use issue and standard-credit denominators, respectively. Minimum-standard obligations are represented by a rating-specific issue share rather than a pooled system percentage:

DCI_d = (I_d/I) × 100(5)

RCS_d = (C_d/ΣC) × 100(6)

MSIS_{d,r} = (m_{d,r}/M_r) × 100(7)

In Equations (1)–(7), d denotes an HST domain and r a BREEAM rating level. For LEED, n_d, P_d and M_d are the domain criterion, point and prerequisite totals; N = 49, ΣP = 110 and M = 16. For BREEAM, I_d and C_d are domain issue and standard-credit totals; I = 51 and ΣC = 165. m_{d,r} is the number of issues in domain d with a minimum at rating r, and M_r is the number of issues with a minimum at that rating. MSIS is undefined when M_r is zero. ΔCW is expressed in percentage points.

Because the complete current YeS-TR office point structure had not been directly extracted from the official annex at the current-data stage, DCI, NWS, and MRD were not calculated as whole-system YeS-TR indicators. Applying system-level metrics to a partial national dataset would produce misleading cross-system comparisons.

### 2.7. Robustness and Multidomain Sensitivity Analysis

To test dependence on secondary assignments, profiles were recalculated under four allocation scenarios: primary-only (1.00/0), 0.75/0.25, 0.50/0.50, and 0.25/0.75 between primary and secondary domains. Items without a secondary code remained fully allocated to the primary domain. For LEED, the same allocation was applied to criterion counts, points, and prerequisites; for BREEAM it was applied to issue counts and standard credits. Rating-specific BREEAM minimum standards remain categorical and are not fractionally allocated.

These scenarios are sensitivity bounds, not claims about the true relative importance of primary and secondary functions. They use the adjudicated secondary assignments made after independent recoding; the initial assignments remain visible in Supplementary Data S1 and the original independent decisions and agreement statistics remain available in Supplementary Data S2. Primary-domain conclusions are supported by independent agreement, but low initial agreement for optional secondary codes limits the reproducibility of fractional profiles. The supplementary workbook reports all four scenarios for both complete systems.

### 2.8. Coding Quality Assurance and Reproducibility

Initial criterion/issue coding was performed by E.Y. using the written HST codebook and a source-page audit trail. M.S.A. independently recoded all 49 LEED criteria and 51 BREEAM issues (100 records), using the same codebook and official sources. He had no prior access to E.Y.’s coding labels and received a version of the dataset with those labels removed for independent recoding. His independent decisions, including those for LEED-1–LEED-5, were recorded before subsequent author discussion. All 100 records were included in initial agreement calculations. Raw agreement and nominal Cohen’s κ were computed separately for primary HST, optional secondary HST with absence treated as a category, and binary presence of a secondary code. After the independent comparison, all 54 optional-secondary differences were reviewed against the written threshold and criterion-level sources; M.S.A. documented decisions and evidence for the remaining 50 after four earlier author decisions. M.S.A. then documented his correction of LEED-27 to no secondary code in a revised adjudication worksheet: resilience is mentioned in the intent, but a separate resilience requirement or verification route is absent. Original labels, returned decisions, corrected adjudication and rationales are separated in Supplementary Data S1 and Supplementary Data S2; initial agreement statistics were not recalculated from reconciled labels. The reconciled secondary codes feed the sensitivity analysis. An intent-based cross-system distinction is retained for BREEAM Pol 03 (HST-9 primary, HST-7 secondary) and LEED Rainwater Management (HST-7 primary, HST-3 secondary), because the latter rewards a separate water-reuse pathway rather than making climate-risk reduction its dominant objective. Of the 54 reconciled differences, 51 final secondary labels corresponded to M.S.A.’s independent labels and 3 did not. Reconciliation was conducted by the authors; it was not adjudication by an independent third party. The corresponding author confirms that M.S.A. had never seen E.Y.’s assigned HST labels before this independent exercise. His validation contribution in the present study consists of independent recoding followed by author review. The supplied decision records document classifications and rationales, but do not constitute an independently timestamped access log.

### 2.9. Matched-Domain Comparison of YeS-TR and LEED v5

The 15 audited YeS-TR criteria were compared with selected LEED v5 conceptual comparators using five attributes: substantive intent, performance or process logic, verification, lifecycle stage, and regulatory function. Each attribute is recorded qualitatively in Supplementary Data S1. “Selected conceptual comparator” indicates an auditable rationale, not a numeric nearest-match algorithm or point equivalence.

Special attention was given to localization. A YeS-TR criterion was interpreted as exhibiting explicit regulatory localization when implementation depended directly on Türkiye-specific certification roles, national reference-building procedures, or national regulatory instruments [28,29].

### 2.10. Documentary Timeline and Source-Control Procedure

The 2020 study was used as a historical documentary reference point. Official regulatory and institutional milestones through 2026 were organized around international-system use, national-system institutionalization, and regulatory integration. This is a documentary timeline analysis, not a longitudinal analysis of repeated measurements or outcomes.

The criterion-level audit trail records source versions, identifiers, HST assignments and explanatory notes. Supplementary Data S1 (Biomimetics_S1_Coding_and_Sensitivity.xlsx) contains the source coding matrices, codebook, YeS-TR audit, rating-specific minima, edition screening and sensitivity calculations. Supplementary Data S2 (Biomimetics_S2_Independent_Recoding.xlsx) contains first-round recoding decisions, agreement calculations and the subsequent disputed-case resolutions. No unverified current point value enters quantitative calculations.

The claims concern formal scoring weights rather than measured building performance; the 15 YeS-TR criteria are not a complete national profile; LEED and BREEAM are compared within their native scoring architectures; the BRL is provisional and cannot establish biomimetic intent; and qualitative localization is not a quantitative fourth score. No system is ranked as superior or inferior.

## 3. Results

### 3.1. Sustainability-Domain Structure of LEED v5 BD+C

The complete LEED v5 BD+C New Construction dataset comprised 49 criteria, including 16 prerequisites and 33 point-bearing credits, with a total of 110 available points. The HST analysis showed a strongly differentiated sustainability structure rather than an even distribution across domains [30].

Energy and Operational Carbon (HST-1) was the dominant domain across all three indicators. It contained 11 of the 49 criteria (DCI = 22.4%), accounted for 29 of the 110 available points (NWS = 26.4%), and included 5 of the 16 prerequisites (MRD = 31.3%). This convergence of criterion density, scoring weight, and mandatory requirements indicates that operational energy and carbon function as the principal structural priority of the current LEED v5 New Construction framework.

Embodied Carbon, Materials and Circularity (HST-2) and Indoor Environmental Quality and Health (HST-5) each contained six criteria (12.2% of the criterion set). HST-2 accounted for 16 points (14.5%), whereas HST-5 accounted for 12 points (10.9%). Transport and Mobility (HST-6) also carried 12 points (10.9%) despite containing only three criteria and no prerequisites, illustrating that criterion count alone does not capture formal scoring emphasis.

The separation of coverage and scoring revealed pronounced structural divergence. Innovation, Regional and Contextual Priorities (HST-10) contained only one primary criterion (DCI = 2.0%) yet carried 9 points (NWS = 8.2%), producing the largest positive coverage-weight gap (ΔCW = +6.14 percentage points). Transport and Mobility (HST-6) likewise had a positive gap of +4.79 points (DCI = 6.1%; NWS = 10.9%). In contrast, Management and Performance Verification (HST-8) had a negative gap of −4.75 points (DCI = 10.2%; NWS = 5.5%), and Social Equity, Accessibility and Community Well-being (HST-11) had a gap of −4.53 points (DCI = 8.2%; NWS = 3.6%). These differences show empirically that the criterion frequency and scoring weight are not interchangeable proxies for certification priority.

Prerequisite density was more concentrated than point allocation. In addition to HST-1, four domains—HST-2, HST-3, HST-5, and HST-8—each contained two prerequisites and therefore represented 12.5% of mandatory requirements. HST-6, HST-7, and HST-10 contained no prerequisites, indicating that their formal influence within LEED v5 operates primarily through optional scoring rather than non-negotiable minimum requirements. The complete LEED domain profile is summarized in Table 2.

**Figure 1:**
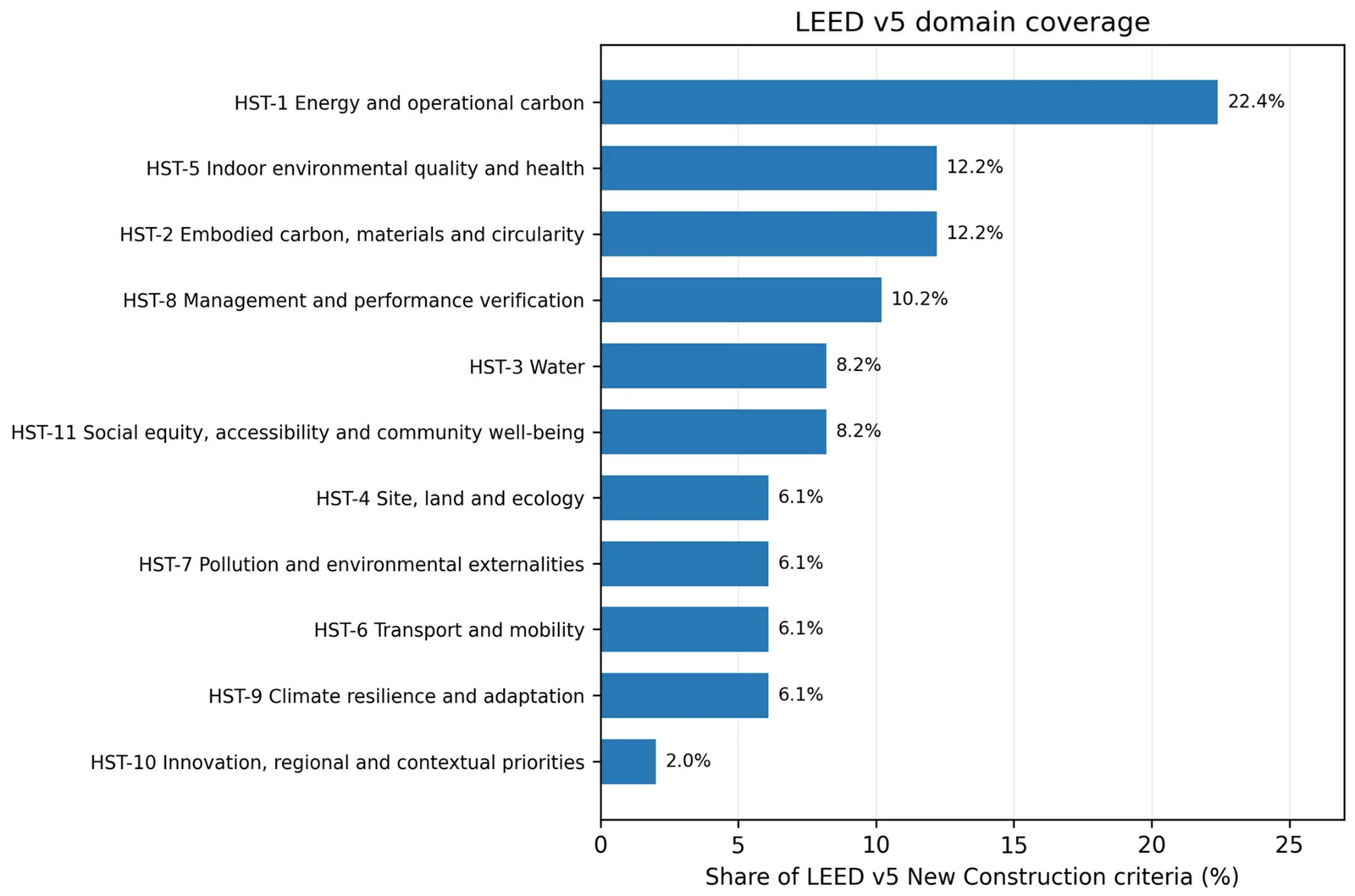
LEED v5 Domain Coverage Index (DCI) by Harmonized Sustainability Taxonomy domain. Note: Percentages are based on the 49 LEED v5 BD+C New Construction prerequisites and credits coded in the study dataset.

**Figure 2:**
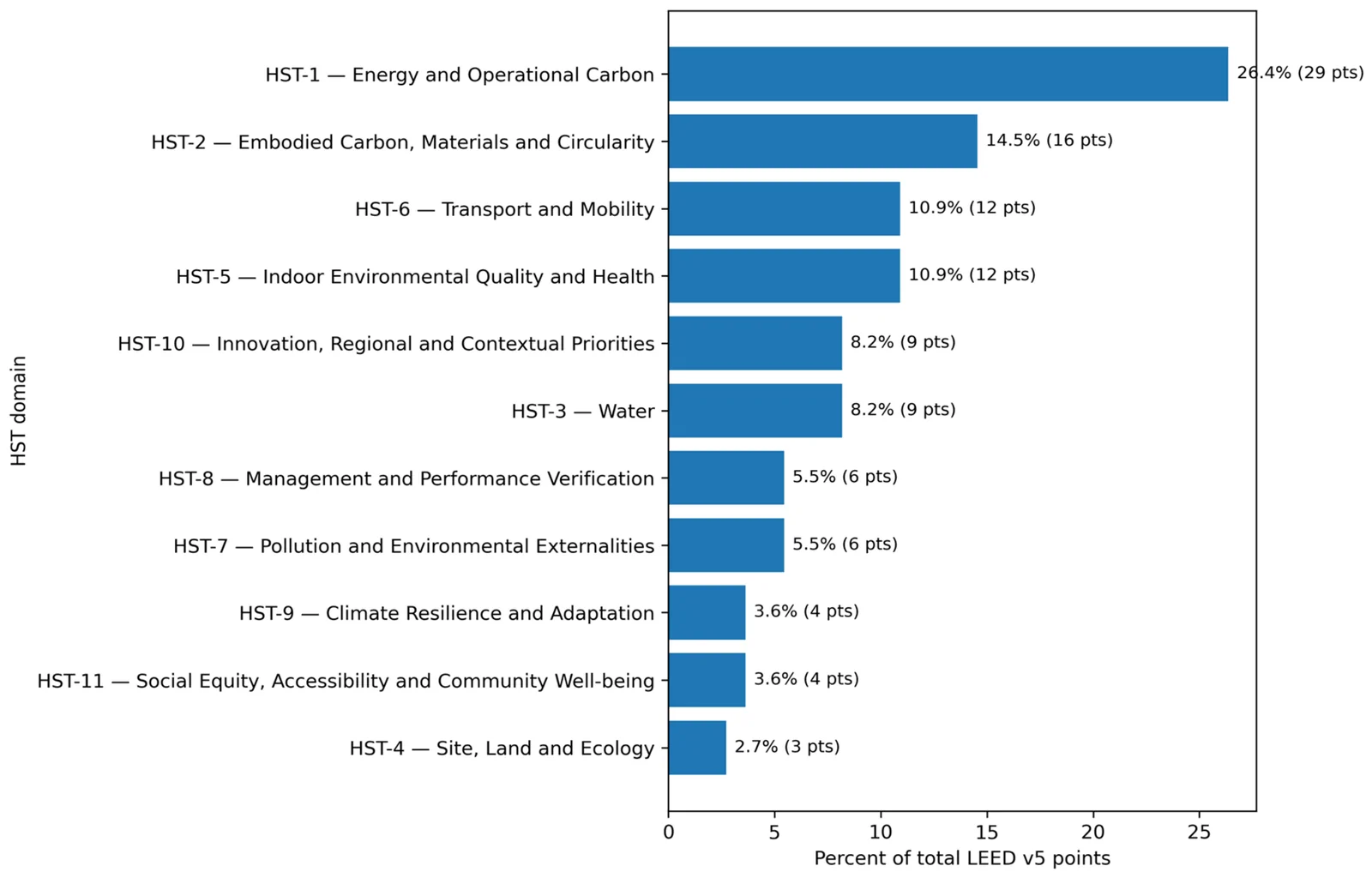
LEED v5 Normalized Weight Share (NWS) by Harmonized Sustainability Taxonomy domain. Note: Percentages are based on the 110 total available LEED v5 points in the New Construction pathway.

**Figure 3:**
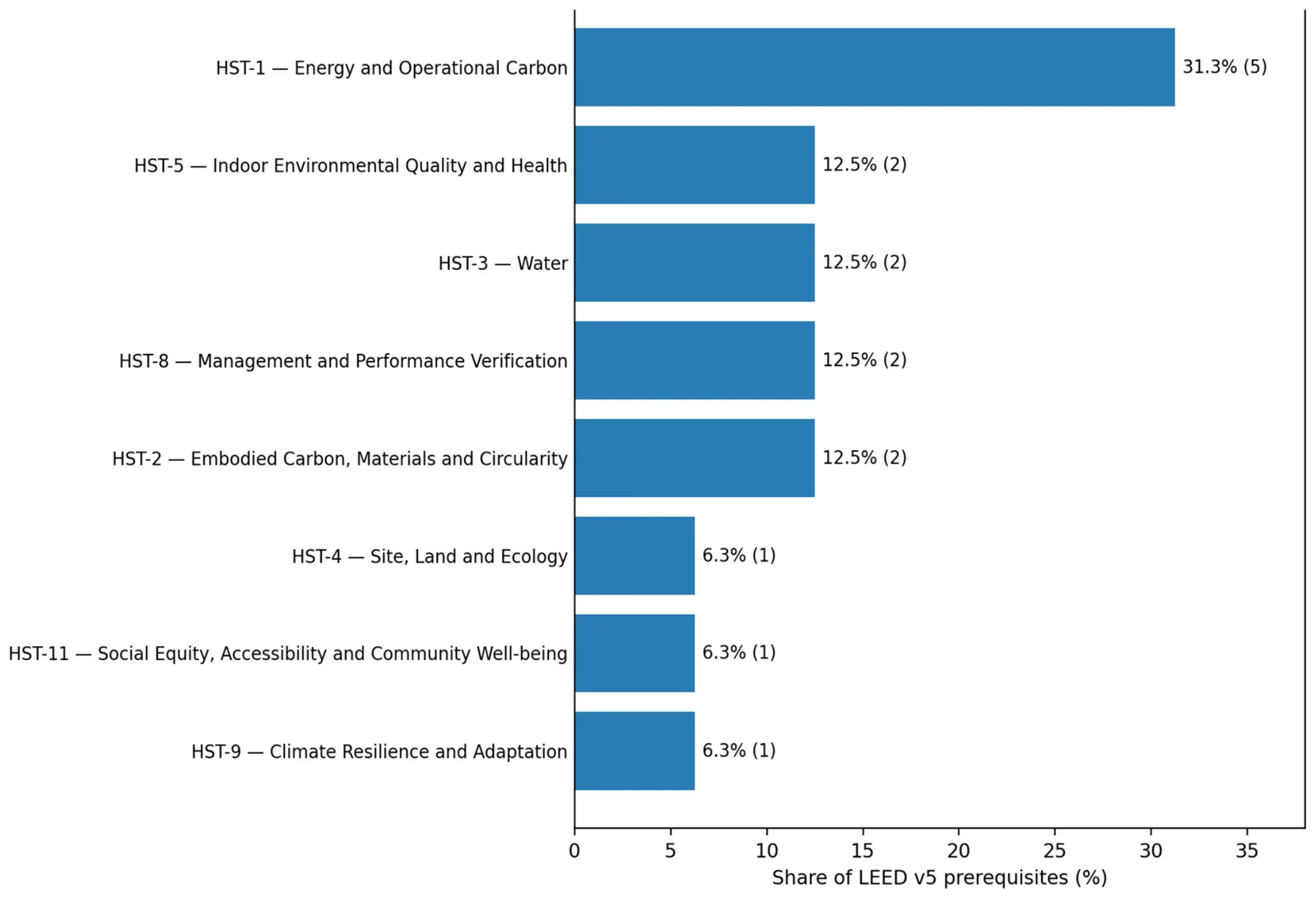
LEED v5 Mandatory Requirement Density (MRD) by Harmonized Sustainability Taxonomy domain. Note: Percentages are based on the 16 LEED v5 prerequisites. Mandatory requirements were analyzed separately from optional scoring weights.

The LEED domain profile is presented visually through criterion coverage, point weighting, and prerequisite density (Figure 1, Figure 2 and Figure 3).

### 3.2. Sustainability-Domain Structure of BREEAM International New Construction V7

The office-applicable BREEAM V7 dataset comprised 51 assessment issues with 165 standard credits. Embodied Carbon, Materials and Circularity (HST-2) contained the largest number of issues (10; DCI = 19.6%), followed by Energy and Operational Carbon (HST-1; 9 issues; 17.6%) and Indoor Environmental Quality and Health (HST-5; 7 issues; 13.7%). In contrast, Energy and Operational Carbon carried the largest share of raw standard credits (49 of 165; RCS = 29.7%), followed by HST-2 (23 credits; 13.9%) and HST-5 and Management/Performance Verification (21 credits each; 12.7%).

The divergence between issue coverage and raw-credit allocation was substantial. HST-1 gained approximately 12 percentage points from issue coverage to raw-credit share, whereas HST-2 showed the reverse pattern. Because Version 7 subsequently applies locally adapted category weights, raw credits represent only one layer of the final scoring architecture [31].

**Figure 4:**
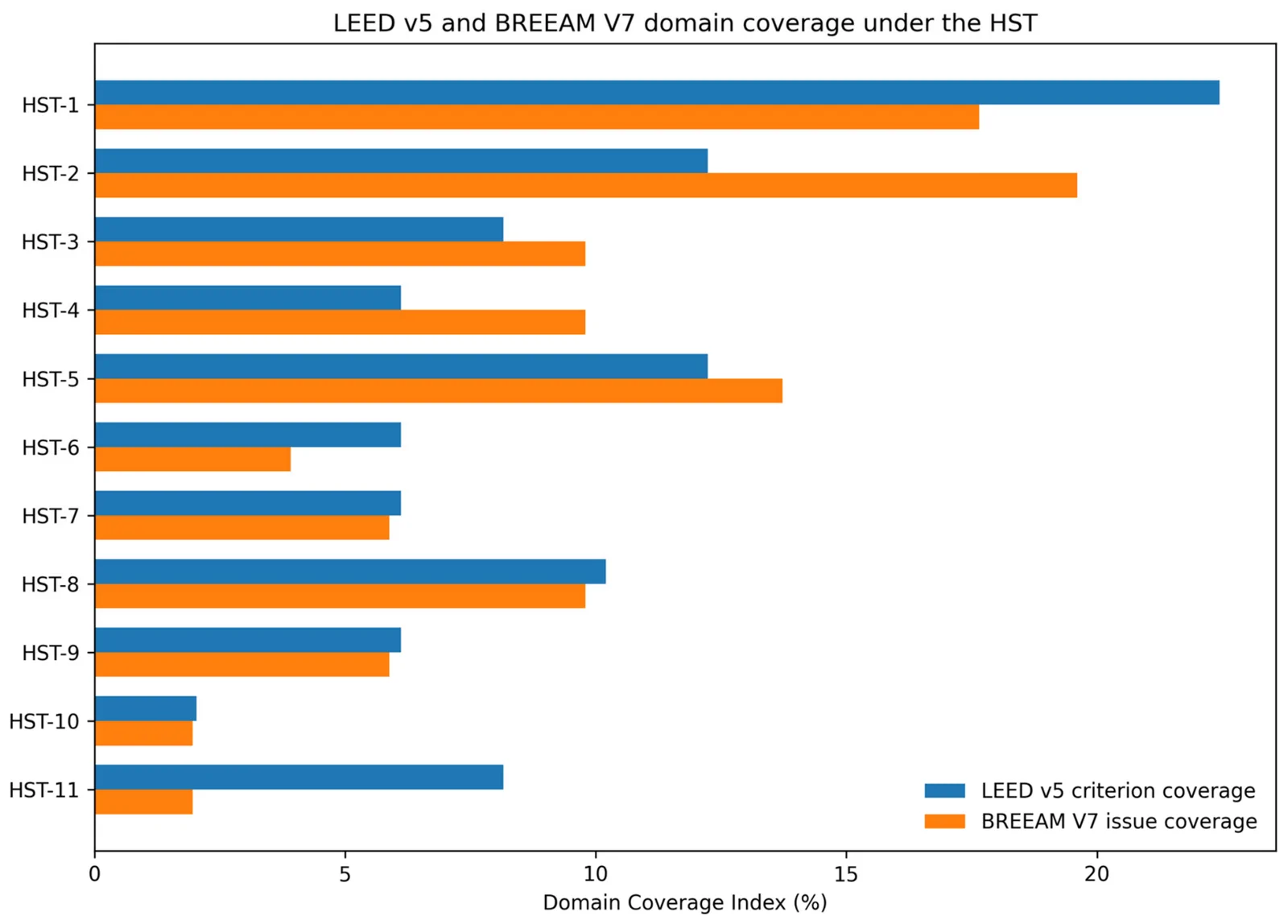
LEED v5 and BREEAM V7 domain coverage under the Harmonized Sustainability Taxonomy. Note: LEED percentages are based on 49 prerequisites/credits; BREEAM percentages are based on 51 office-applicable assessment issues. Domain key: HST-1 energy/carbon; HST-2 embodied carbon/materials; HST-3 water; HST-4 site/ecology; HST-5 indoor environmental quality; HST-6 transport/mobility; HST-7 pollution/externalities; HST-8 management/verification; HST-9 climate resilience; HST-10 innovation/context; HST-11 social equity/accessibility.

**Figure 5:**
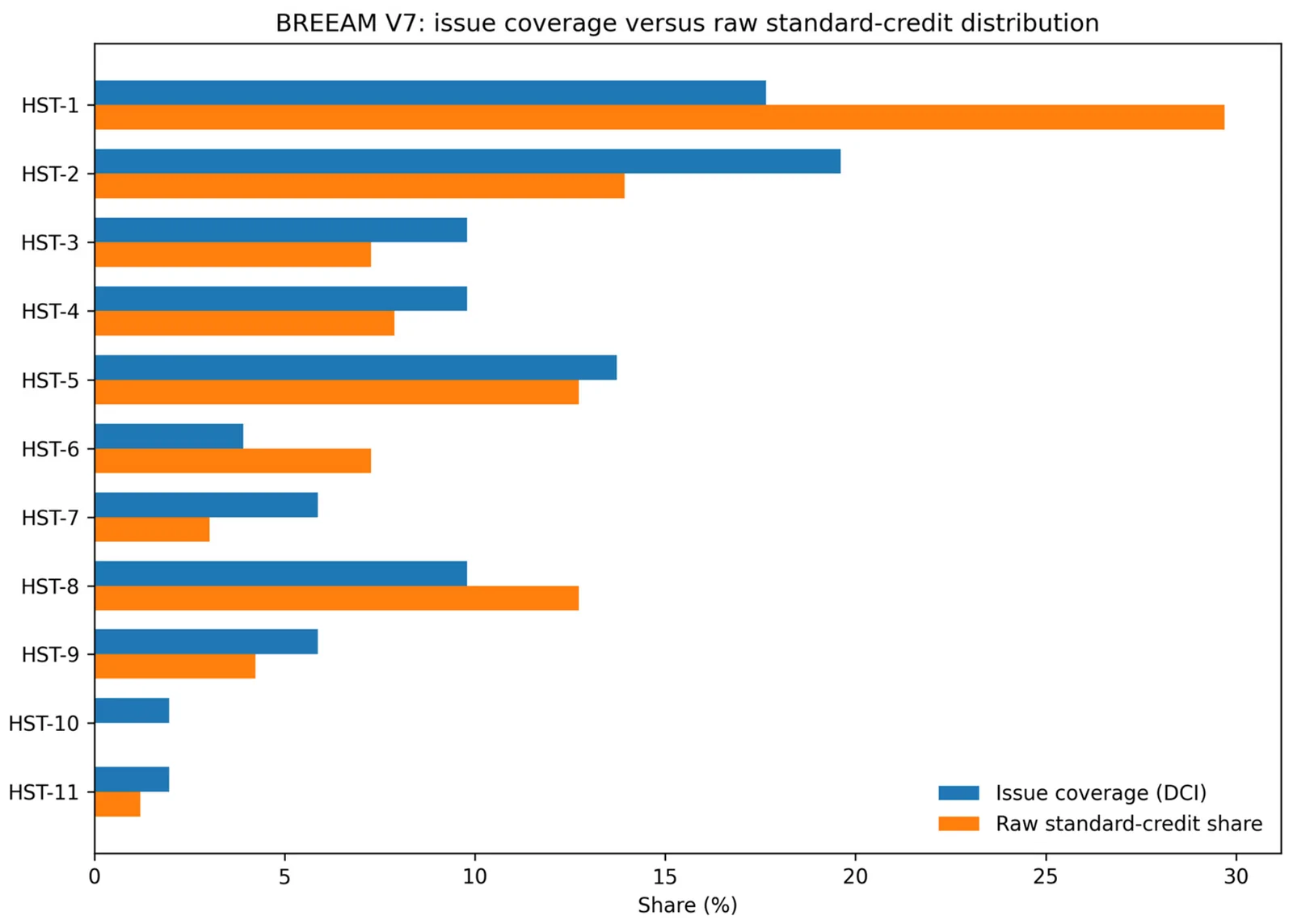
BREEAM V7 issue coverage and raw standard-credit distribution by HST domain. Note: Raw standard-credit shares precede BREEAM’s country-specific category weighting and therefore do not represent the final weighted BREEAM score. Domain key: HST-1 energy/carbon; HST-2 embodied carbon/materials; HST-3 water; HST-4 site/ecology; HST-5 indoor environmental quality; HST-6 transport/mobility; HST-7 pollution/externalities; HST-8 management/verification; HST-9 climate resilience; HST-10 innovation/context; HST-11 social equity/accessibility.

Sixteen office-applicable issues were linked to at least one rating-dependent minimum standard. Because minima vary by target rating, a pooled share would obscure differences in when an obligation applies. The issue-by-rating matrix is reported in Supplementary Data S1. Table 3 summarizes primary-domain profiles; Figure 4 and Figure 5 visualize coverage and the BREEAM coverage–credit relationship.

### 3.3. Cross-System Structural Comparison: Fixed Points Versus Weighted Scorecards

LEED v5 and BREEAM V7 showed substantive convergence but different scoring architectures. Energy/Operational Carbon was prominent in both systems; however, LEED combines fixed points and prerequisites, whereas BREEAM combines raw credits, locally adaptable category weighting, and rating-dependent minimum standards. Materials/Circularity was more prominent in BREEAM issue coverage (19.6%) than in LEED criterion coverage (12.2%), while Social Equity/Accessibility was more visible as a primary LEED domain than as a primary BREEAM issue domain. The BREEAM social-domain result is based on one primary-coded issue, so this contrast is descriptive and sensitive to issue granularity.

These results demonstrate why point totals should not be compared directly. LEED criteria and BREEAM assessment issues are different native units, and an issue can bundle more subrequirements than a LEED criterion; DCI shares therefore retain a residual granularity bias even when both denominators are complete.

### 3.4. Audited Matched-Domain Comparison of YeS-TR and LEED v5

The purposive YeS-TR audit included 15 criteria: seven mandatory and eight credit-bearing or optional. The criteria are distributed across four categories and were selected for source verifiability and conceptual matching; their frequencies are not interpreted as system-wide emphasis. BBT 01 K4 combines site selection and transport connections. Site/mobility was therefore considered through this bundled item only; the audit does not establish complete YeS-TR mobility-domain coverage.

The matched-domain analysis revealed substantial conceptual overlap but different modes of operationalization. In integrated project governance, YeS-TR makes formation of the interdisciplinary project team (BBT 01 K1), detailed project-scope definition (BBT 01 K3), and interdisciplinary stakeholder participation (BBT 02 K1) mandatory. These requirements correspond conceptually with LEED v5 Integrative Design Process and professional integration mechanisms, but YeS-TR expresses them through nationally defined project roles and procedural obligations [29,30].

Indoor environmental quality provided the clearest cluster of directly comparable technical requirements. YeS-TR makes the required illuminance level (İOK 01 K1) and outdoor-air provision for the office/non-residential pathway (İOK 04 K1) mandatory, while daylight performance (İOK 01 K5), color rendering (İOK 01 K4), and the office-pathway Predicted Mean Vote/Predicted Percentage Dissatisfied (PMV/PPD) thermal-comfort criterion (İOK 03 K1) operate as credit-bearing requirements in the audited dataset. LEED v5 similarly combines baseline air-quality prerequisites with point-bearing occupant-experience and enhanced-air-quality credits, but it groups several dimensions of visual and thermal experience within broader composite credits [29,30].

The audited energy criterion EKV 01 K1 links minimum energy performance to Türkiye’s national reference-building and Türkiye’s Building Energy Performance Regulation system (BEP-TR) framework. This is a descriptive example of regulatory localization in the selected audit set; one criterion does not establish that energy has the strongest localization across YeS-TR.

Water showed a related but structurally different pattern. YeS-TR SAY 01 K1 addresses efficient fixture and fitting selection relative to a reference-building improvement approach and was coded as a credit in the audited office pathway. LEED v5 separates minimum and enhanced water performance through a prerequisite-credit sequence (WEp2 and WEc2). The comparison therefore suggests similar performance objectives but different packaging of baseline and enhanced requirements [29,30].

YeS-TR BBT 04 K3 requires construction worker health and safety. Its selected LEED comparator, EQp1 Construction Management, has broader construction-phase environmental and air-quality objectives; the match is partial and does not establish equivalence. The YeS-TR item is coded primarily to HST-11, while the LEED prerequisite is coded to HST-8 because of its management and verification structure. This divergence is retained and limits the strength of the cross-system comparison [29,30].

The audited comparisons identify national professional roles, project processes and reference-building procedures alongside selected international comparators. They support descriptive comparisons of implementation mechanisms, without whole-system YeS-TR weighting or ranking. Table 4 summarizes the matches and their limits.

### 3.5. Documentary National Context

The 2020 study provides a historical reference context [23]. National implementation procedures and the 2022 Green Certificate Regulation subsequently formalized YeS-TR [34,35]. The current timeline documents legal and institutional milestones rather than repeated measurements of certification use or building performance.

**Figure 6:**
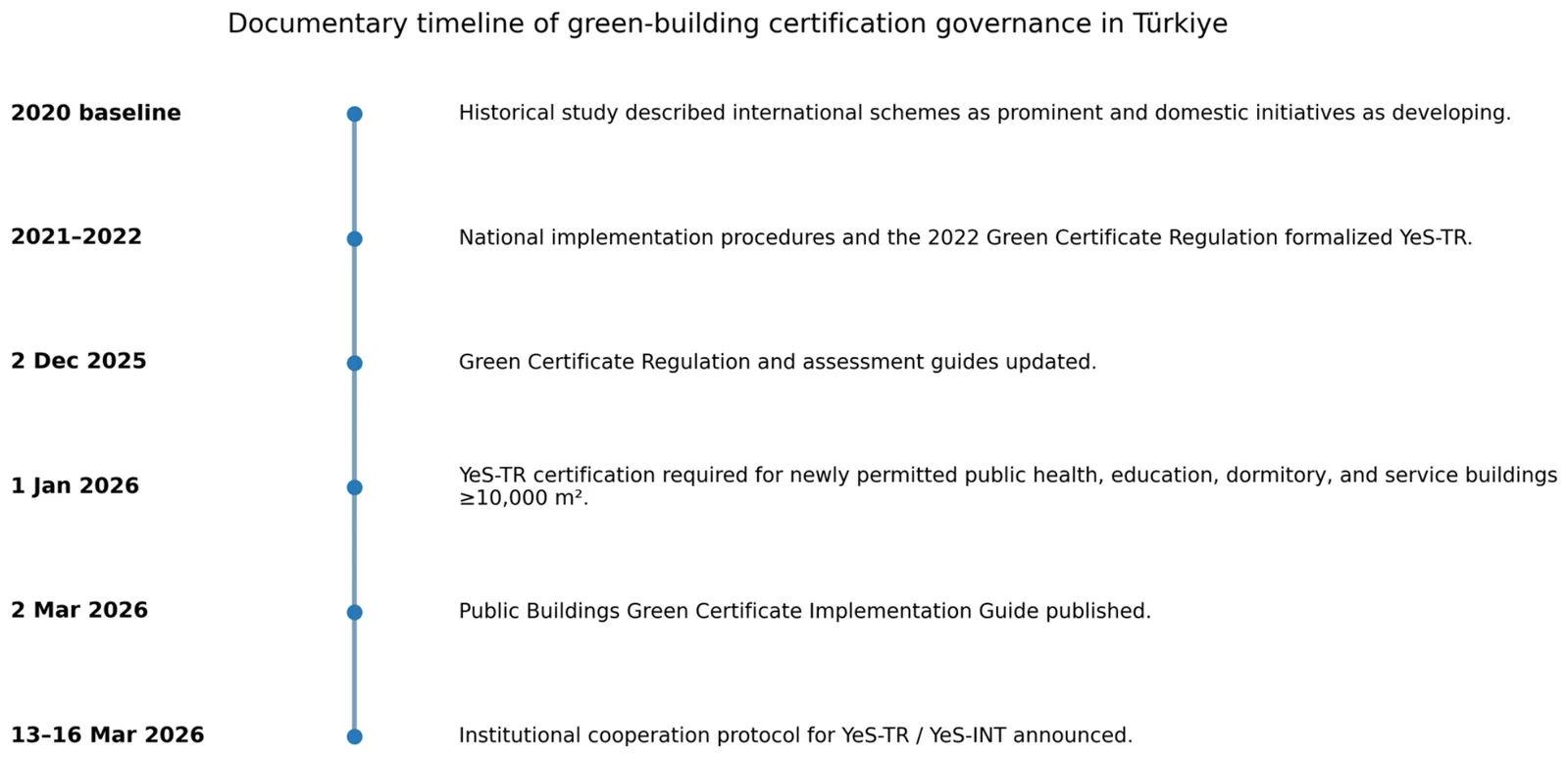
Verified milestones in the evolution of green building certification in Türkiye (2020–2026). Note: The timeline reflects only milestones verified through the cited official and scholarly sources and does not imply causality regarding certification uptake.

The assessment framework was updated on 2 December 2025. The 11 March 2025 zoning amendment (Official Gazette No. 32838) requires certification from 1 January 2026 for newly permitted public health, education, dormitory and service buildings with a total construction area of at least 10,000 m^2^ [24]. The March 2026 implementation guide and cooperation announcement complete the selected timeline [28,29,36]. Table 5 and Figure 6 summarize these milestones; effects on uptake, costs and building outcomes were not measured.

Figure 6 visualizes the documentary timeline, including the 2021–2022 institutionalization milestone listed in Table 5.

### 3.6. Sensitivity and Structural Robustness of LEED and BREEAM Profiles

Under primary-only allocation, LEED HST-1 accounted for 22.45% of criteria, 26.36% of points, and 31.25% of prerequisites. With the reconciled secondary codes, the 0.75/0.25, 0.50/0.50, and 0.25/0.75 scenarios assign HST-1 criterion shares of 21.43%, 20.41%, and 19.39%; point shares of 26.82%, 27.27%, and 27.73%; and prerequisite shares of 28.13%, 25.00%, and 21.88%, respectively. HST-1 remains the largest point-share domain. The four scenarios for LEED and BREEAM are in Supplementary Data S1.

Under the reconciled 0.50/0.50 allocation, the LEED HST-11 point share changes from 3.64% to 5.45%, HST-4 from 2.73% to 6.36%, and HST-9 from 3.64% to 5.00%. HST-8 criterion coverage increases from 10.20% to 15.31%. In the original independent recoding, primary codes matched on all 100 records (100%; Cohen’s κ = 1.000). Optional secondary codes matched on 46 of 100 (46.00%; κ = 0.350), counting absence as a category; presence/absence matched on 51 of 100 (51.00%; κ = 0.182). E.Y. initially assigned a secondary code to 81 items and M.S.A. to 34. All 54 initial differences were then adjudicated, leaving 34 of 100 items with a reconciled secondary code (LEED 24/49; BREEAM 10/51). Adjudication does not change the initial independent reliability estimates or establish biological multifunctionality.

The four allocation weights are scenarios rather than validated estimates. Table 6 presents the reconciled 0.50/0.50 LEED scenario; all four reconciled scenarios for LEED and BREEAM are in Supplementary Data S1. The lower incidence after source-based adjudication shows that the original fractional redistribution was sensitive to coder interpretation. Initial optional-secondary agreement remains limited, so these scenarios should be interpreted as exploratory rather than independently replicated measurements.

**Figure 7:**
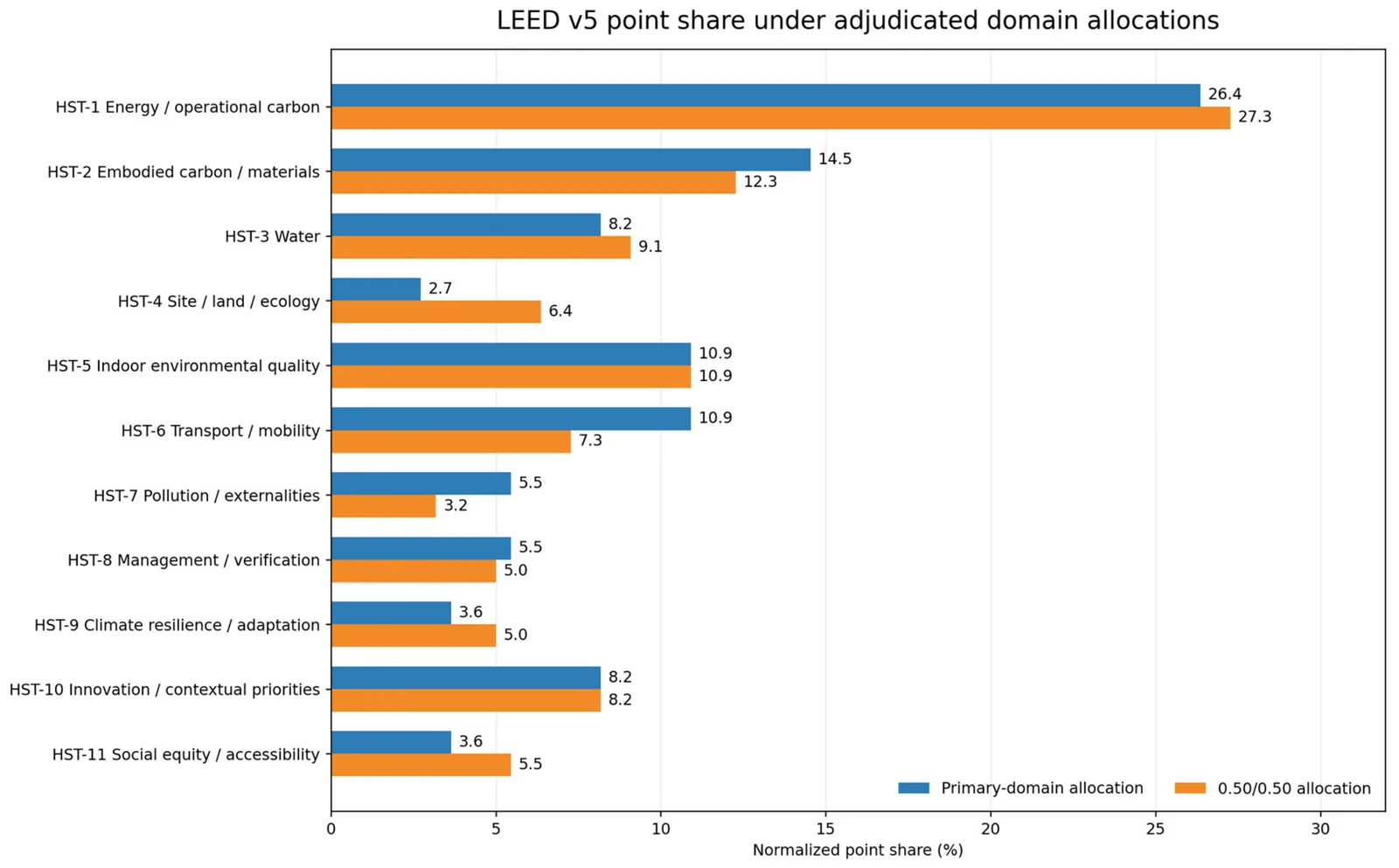
LEED v5 normalized point shares under primary-only and 0.50/0.50 allocation using reconciled optional secondary HST codes. All four LEED and BREEAM scenarios are in Supplementary Data S1. Note: Primary-domain allocation assigns each credit entirely to its primary HST domain. Fractional allocation divides credits equally between primary and secondary HST domains when both are coded. Domain key: HST-1 energy/carbon; HST-2 embodied carbon/materials; HST-3 water; HST-4 site/ecology; HST-5 indoor environmental quality; HST-6 transport/mobility; HST-7 pollution/externalities; HST-8 management/verification; HST-9 climate resilience; HST-10 innovation/context; HST-11 social equity/accessibility.

Figure 7 visualizes the reconciled 0.50/0.50 LEED point-share scenario. The full four-scenario output for both complete systems is available in Supplementary Data S1.

### 3.7. Provisional Biomimetic Readiness Lens Profiles

Under the provisional BRL mapping, the primary-domain profile groups HST-1 to HST-3 as resource frugality/circularity (42.9% of LEED criteria; 47.1% of BREEAM issues). Ecological integration/externalities (HST-4 and HST-7) account for 12.2% and 15.7%, respectively. Climate adaptation/resilience (HST-9 alone) accounts for 6.1% and 5.9%. Network/mobility (HST-6) accounts for 6.1% and 3.9%; coordination/verification (HST-8), 10.2% and 9.8%; and social cooperation/well-being (HST-11), 8.2% and 2.0%. HST-5 and HST-10 remain unmapped. These are regrouped sustainability-coverage shares, not biomimetic-readiness scores.

The reconciled optional secondary codes produce smaller fractional shifts than the original coder’s labels, but allocation assumptions still change the grouped profiles. Supplementary Data S1 (BRL_Adjudicated_Scenarios) reports primary-only, 0.75/0.25, 0.50/0.50, and 0.25/0.75 count- and point/raw-credit-weighted BRL profiles for both complete systems. BRL-1 remains the largest dimension under each allocation and weighting within each system; smaller dimension shares and their ordering vary. Because HST-5 and HST-10 are intentionally unmapped, the six BRL shares do not sum to 100% of all criteria or credits. Secondary-domain incidence is not interpreted as evidence of biological multifunctionality.

The YeS-TR audit is not included in Table 7 or in the BRL profile comparison. Its 15 criteria were purposively selected from four categories and cannot support a national system profile.

Reconciled secondary-domain assignments are summarized by the HST domain in Supplementary Data S1, with first-round E.Y. and M.S.A. labels retained separately. Their incidence is not treated as a validated measure of biological multifunctionality or used for a system ranking. Supplementary Data S2 records the 54 initial differences, evidence and subsequent decisions.

### 3.8. Quantitative Profiles and Qualitative Localization

Coverage, scoring reward and requirements use system-specific quantitative indicators; localization is qualitative. RQ1 is addressed in Section 2.5 and Section 3.7; RQ2 in Section 3.1, Section 3.2 and Section 3.3; RQ3 in Section 3.4; RQ4 in Section 3.6; RQ5 in Section 3.5; and RQ6 in Section 3.4 and Section 4.2. These layers describe different properties and are not combined into a ranking.

Source-control statement. All criterion-level claims and quantitative calculations in Section 2 and Section 3 are based on the primary or official institutional sources cited in the text. Where a current primary-source value could not be directly verified, it was excluded from quantitative calculation rather than inferred from secondary reproductions.

## 4. Discussion

### 4.1. Certification Architecture and the Limits of Cross-System Comparison

The distinction between DCI, NWS, and MRD is not only a reporting choice but also a substantive methodological finding. In LEED v5, the largest coverage–weight gaps occurred in HST-10 (+6.14 percentage points) and HST-6 (+4.79), showing that a small number of high-value credits can create strong scoring leverage, while HST-8 (−4.75) and HST-11 (−4.53) demonstrate the reverse pattern: broader criterion presence can coexist with limited optional point weight. BREEAM V7 provides an independent structural analogue. Materials/Circularity had the greatest issue coverage (19.6%), yet Energy/Operational Carbon carried the largest raw standard-credit share (29.7%). The 16 office-applicable BREEAM issues with minimum-standard provisions apply at different rating levels. Their issue-by-rating matrix is reported in Supplementary Data S1; a pooled domain share is not treated as comparable with LEED prerequisites, which apply at every certification level. Thus, both international systems demonstrate that issue prevalence, scoring leverage, and minimum-performance obligations can diverge, even though the mechanisms through which they do so are different. Comparative studies that rely on a single normalized percentage therefore risk conflating distinct governance mechanisms [8,17,31].

The prominence of operational energy and carbon is consistent with recent carbon-focused GBRS research. Olanrewaju et al. [14] reported that operational credits substantially exceed embodied credits across several systems, and Ng et al. [15] similarly identified persistent operational-carbon dominance and fragmented life-cycle treatment. Arslan and Ulubeyli [16] found strong whole-life-carbon alignment for both LEED and YeS-TR, but the present analysis adds a different layer: within LEED v5, energy/carbon dominance persists across coverage, points, prerequisites, and the multidomain sensitivity test. This robustness strengthens the interpretation of HST-1 as a structural priority, while the study still distinguishes formal certification architecture from measured building performance. Empirical evidence on LEED-certified buildings remains mixed, so NWS and MRD should not be read as proxies for realized operational savings [37,38].

### 4.2. YeS-TR Localization in the Audited Criteria

The 15-criterion audit illustrates national implementation mechanisms through project-team roles, stakeholder participation, reference-building energy assessment and worker protection. These examples complement the Turkish certification literature [16,20,21,22] without quantifying whole-system localization. EKV 01 K1 links minimum energy performance to BEP-TR; this single example does not establish the strongest localization domain. The partial worker-protection match and bundled site/transport item remain qualified in Section 3.4 and Table 4.

### 4.3. Documentary Governance Timeline

The selected milestones document the formal institutional context [23,24]; they supply no uptake or market-displacement measurements. Their role is to explain the national setting of the bounded audit. They are not a basis for interpreting BRL coverage as a biological or design outcome.

### 4.4. Methodological Implications and the Provisional BRL

The HST-based procedure has methodological implications beyond the Türkiye case. Many comparative GBRS studies normalize original categories into common themes, yet comparisons can remain distorted if prerequisites, optional points, raw credits, category weights, and rating-dependent minimum standards are treated as interchangeable measures. The present framework therefore separates the architecture into four questions: what a system covers, what it rewards, what it requires, and how it localizes sustainability objectives. DCI, NWS, MRD, BREEAM RCS/MSIS, the coverage–weight gap, matched-domain analysis, and multidomain sensitivity are not proposed as a single composite score; they are complementary diagnostics designed to preserve the native logic of each certification system while still enabling transparent comparison. This is the study’s principal generalizable methodological contribution.

The primary-only profiles show Energy and Operational Carbon as a leading domain, and the independent recoding agrees on all eligible primary labels. Source-based adjudication substantially narrows the optional secondary layer to 34 of 100 items; 0.50/0.50 allocation therefore produces smaller shifts than the original coding. The 46/100 initial exact matches nevertheless limit claims of independent reproducibility, and the reconciled scenarios do not establish biological multifunctionality.

The matched-domain procedure provides a second safeguard. Instead of assuming that similarly named categories are equivalent, criteria were matched according to substantive intent, performance/process logic, verification mechanism, lifecycle stage, and regulatory function. This is consistent with criteria-based comparison approaches used in prior GBRS research [9], while extending them through explicit source-control boundaries. In particular, the analysis does not generate a whole-system YeS-TR score from a partial dataset. This conservative treatment reduces apparent comprehensiveness but increases reproducibility and protects the interpretation from unsupported rankings.

### 4.5. Implications for Certification Development

National certification developers can use the audited criteria to review professional responsibilities, reference-building procedures, and public-sector compliance routes. These are specific institutional design questions; their effectiveness requires subsequent implementation evidence.

Certification architecture should be reviewed through separate coverage, reward and requirement indicators. The LEED coverage–weight gaps show that a high point share can coexist with narrow criterion coverage, while prerequisites can give importance to a domain with limited elective points. Any benefit from changing professional roles or national implementation procedures requires subsequent implementation evidence.

### 4.6. Implications for Biomimetic Research

The BRL is an exploratory interpretive device for asking how conventional sustainability coverage can be grouped under selected bio-inspired functional themes. Its value lies in making that crosswalk visible: for example, a reader can distinguish circular resource criteria from resilience, mobility, coordination, or social criteria, even when the source systems use unrelated categories. This regrouping can guide questions for future biomimetic research, but it does not test whether a criterion or building is biomimetic, nor whether certification supports or constrains design decisions.

Optional secondary assignments require cautious interpretation. The 54 initial differences document low reproducibility of this optional layer. Subsequent source-based adjudication left 34 of 100 items with a secondary code but cannot retrospectively improve initial inter-coder agreement or establish biological multifunctionality. Future work should test the rule with additional independent coders and trace biological models and design outcomes at the project level. The single optional-secondary field also limits the representation of multiple functions: an item with several additional functions can receive only one secondary label, and no function-strength score was collected.

The comparatively small primary shares for adaptation, networks, and social cooperation suggest candidate areas for further investigation, not demonstrated system deficiencies. Relevant examples include climate-resilience criteria (HST-9), transport/mobility criteria (HST-6), and explicit accessibility or workforce/community provisions (HST-11). Whether additional sensing, feedback, mutualism, or adaptive-control requirements would improve biomimetic design support remains a hypothesis requiring project-level evidence.

### 4.7. Limitations and Future Research

Several limitations define the scope of the findings. First, the quantitative system-level analysis is complete for LEED v5 BD+C but not for YeS-TR. The 15 YeS-TR criteria constitute an audited analytical subset selected to test multiple domains; they are not a statistically representative sample of the entire national rating system. Consequently, no whole-system YeS-TR DCI, NWS, or MRD values are reported, and no overall ranking between LEED v5 and YeS-TR is attempted.

The BRL is a provisional deterministic mapping and has no external construct validation. It cannot distinguish biomimetic readiness from sustainability coverage and omits functions not represented in HST, including life-friendly chemistry, self-assembly, form-based passive adaptation, and ecological mutualism as distinct from site/ecology protection. Future testing should compare the lens with established biological-principle frameworks, expert review, biological-model tracing, and known-groups cases that include explicitly biomimetic or regenerative schemes. Until then, BRL profiles should be described as exploratory regroupings.

Initial coding was conducted by E.Y.; M.S.A. independently coded all 100 records, including five LEED decisions the corresponding author confirmed had been saved before later discussion. Primary-code agreement was complete, but initial optional secondary agreement was limited (46/100 exact matches, κ = 0.350), and the coders initially assigned secondary domains at different rates (81 versus 34 of 100). All 54 initial optional-secondary differences were subsequently adjudicated against criterion-level source evidence; the reconciled dataset assigns a secondary code to 34 items. The original labels and agreement statistics are preserved separately from the reconciled sensitivity outputs. This reliability check concerns HST assignments, not BRL construct validity or building performance outcomes. Final labels followed the second coder’s independent classification in 51 of the 54 disputed cases. This author-led reconciliation does not constitute an additional independent reliability test.

BREEAM V7 was analyzed from the official technical manual, but no Türkiye-specific category-weight set was available in the consulted source. The study therefore reports issue coverage, raw standard-credit allocation, and rating-dependent minimum-standard matrices. The YeS-TR audit is purposive, and selection documentation is incomplete: no full candidate count or exclusion log was retained. These limitations reduce reproducibility and preclude inference about whole-system YeS-TR emphasis. B.E.S.T.-Ticari is contextual only.

Fourth, the present study evaluates the certification architecture and governance rather than measured building outcomes. Formal credit structures cannot demonstrate whether certified buildings achieve predicted energy, carbon, water, or occupant-performance outcomes in operation. The mixed empirical evidence on certified-building energy performance underscores the need for future Turkish research linking YeS-TR certification records with post-occupancy utility, indoor-environmental, and user data [37,38].

The regulatory timeline is documentary and ends in 2026. It does not measure repeated observations, adoption, compliance costs, certification outcomes, or causal effects. Future research should evaluate those outcomes as implementation evidence accumulates.

## 5. Conclusions

This study compares the formal architecture of LEED v5 BD+C and BREEAM International New Construction V7, adds a purposive audit of selected YeS-TR criteria, and summarizes a documentary timeline of certification governance in Türkiye. It distinguishes formal coverage, scoring incentives, minimum requirements, and qualitative localization while keeping certification structures separate from measured building outcomes.

The international-system results distinguish coverage, reward, and requirement. In LEED v5, Energy and Operational Carbon dominated criterion coverage (22.4%), normalized point share (26.4%), and prerequisite density (31.3%). In BREEAM V7, Materials/Circularity had the greatest issue coverage (19.6%), whereas Energy/Operational Carbon had the greatest raw standard-credit share (29.7%); 16 office-applicable issues were linked to rating-dependent minimum standards. These findings show that breadth, scoring leverage, and minimum-performance obligations are structurally distinct even within mature international certification systems.

Alternative allocation scenarios show how the reconciled secondary assignments redistribute domain shares. The initial reliability check supports the primary classification but found limited optional secondary agreement; adjudication does not alter that result. Readers should prioritize primary-domain profiles and treat the reconciled four-scenario analyses in Supplementary Data S1 as exploratory bounds. The item-level initial differences and adjudication decisions are available in Supplementary Data S2.

The YeS-TR audit documents examples of national regulatory embedding in selected criteria. It cannot establish whole-system emphasis because criteria were purposively selected. The 2020–2026 timeline records policy and institutional milestones, not repeated outcome measurements.

The BRL offers an exploratory crosswalk that makes the HST domains interpretable under selected bio-inspired functional themes. It adds a regrouped view of existing sustainability coverage, but no new observations. The study does not demonstrate biomimetic readiness, biological multifunctionality, or that certification supports or constrains design. Those claims require external validation and project-level evidence.

Future work should independently replicate the source-based secondary-code rule, complete a documented YeS-TR criterion audit, test the BRL against established biological-principle frameworks and known-groups cases, and link certification criteria to design decisions and post-occupancy outcomes.

## Figures and Tables

**Table 1:** Biomimetic Readiness Lens (BRL): operational dimensions and HST mapping.

BRL Dimension	Bio-Inspired Functional Interpretation	Mapped HST Domains	Operational Use	Biological Principle Basis and Source
BRL-1 Resource frugality and circular flows	Resource demand reduction, reuse and cycling	HST-1, HST-2, HST-3	Regrouped primary-domain coverage; not a biological function score	Resource efficiency: energy-conserving processes and recycling of materials; resource-flow analogy only [33].
BRL-2 Ecological integration and externalities	Site/ecology protection and pollution/externality reduction	HST-4, HST-7	Regrouped primary-domain coverage	Local responsiveness and benign chemical outputs; habitat protection or pollution control alone does not establish those biological mechanisms [33].
BRL-3 Climate adaptation and resilience	Explicit climate adaptation or resilience requirements	HST-9 only	HST-5 excluded: occupant conditions alone do not demonstrate adaptation	Adaptation through diversity, renewal, variation, redundancy and decentralization; climate-risk requirements provide a limited analogy [33].
BRL-4 Network and mobility	Movement and transport connectivity	HST-6	Regrouped primary-domain coverage	Growth through modular organization across nested scales; transport connectivity is an analogy, not proof of a biological network [33].
BRL-5 Coordination and verification	Management, commissioning, and performance verification	HST-8	No biological feedback inference without a monitoring-response loop	Local responsiveness through feedback; coordination and verification are not evidence of feedback without a monitoring-response loop [33].
BRL-6 Social cooperation and well-being	Accessibility, workforce/community well-being and equity	HST-11	Regrouped primary-domain coverage	Local responsiveness through cooperative relationships; accessibility and social equity are not proof of reciprocal ecological cooperation [33].

Note: BRL is an unvalidated, second-order interpretation of HST coverage, not a measure of biological imitation, biomimetic intent, or design readiness. HST-5 and HST-10 are not mapped; see Methods. The principle column provides a conceptual basis for the provisional labels, not evidence of construct validation. Conventional HST coverage does not establish biological emulation, an operating feedback loop, or reciprocal ecological benefit.

**Table 2:** LEED v5 BD+C sustainability-domain profile based on the Harmonized Sustainability Taxonomy.

HST Domain	Criteria, *n*	DCI (%)	Points	NWS (%)	Prerequisites, *n*	MRD (%)
HST-1	11	22.4	29	26.4	5	31.3
HST-2	6	12.2	16	14.5	2	12.5
HST-3	4	8.2	9	8.2	2	12.5
HST-4	3	6.1	3	2.7	1	6.3
HST-5	6	12.2	12	10.9	2	12.5
HST-6	3	6.1	12	10.9	0	0.0
HST-7	3	6.1	6	5.5	0	0.0
HST-8	5	10.2	6	5.5	2	12.5
HST-9	3	6.1	4	3.6	1	6.3
HST-10	1	2.0	9	8.2	0	0.0
HST-11	4	8.2	4	3.6	1	6.3
Total	49	100.0	110	100.0	16	100.0

Note: DCI = Domain Coverage Index; NWS = Normalized Weight Share; MRD = Mandatory Requirement Density. Percentages may not sum exactly because of rounding. Source: Authors’ calculations based on the LEED v5 BD+C February 2026 rating-system documentation [30].

**Table 3:** BREEAM International New Construction V7 office-applicable sustainability-domain profile.

HST Domain	Issues, *n*	DCI (%)	Standard Credits	RCS (%)	Issues with Any Rating-Specific Minimum, *n*
HST-1	9	17.6	49	29.7	4
HST-2	10	19.6	23	13.9	4
HST-3	5	9.8	12	7.3	2
HST-4	5	9.8	13	7.9	0
HST-5	7	13.7	21	12.7	3
HST-6	2	3.9	12	7.3	0
HST-7	3	5.9	5	3.0	0
HST-8	5	9.8	21	12.7	3
HST-9	3	5.9	7	4.2	0
HST-10	1	2.0	0	0.0	0
HST-11	1	2.0	2	1.2	0

Note: DCI = Domain Coverage Index; RCS = Raw Credit Share. RCS uses 165 standard credits before country-specific weighting. Minimum standards vary by rating level; see the issue-by-rating matrix in Supplementary Data S1. The single BREEAM HST-10 issue has zero standard credits and remains in the issue-count denominator; excluding it changes the denominator to 50 but leaves the primary coverage leader (HST-2) and credit-share leader (HST-1) unchanged. LEED’s HST-10 innovation credit contributes 9 points; excluding it changes the point denominator to 101, with HST-1 remaining the largest point domain (29/101 = 28.7%). Exemplary/innovation credit data are documented separately in Supplementary Data S1 and excluded from standard-credit shares. Source: [31].

**Table 4:** Summary of the audited YeS-TR–LEED v5 matched-domain comparison.

Comparison Dimension	YeS-TR Audited Evidence	Closest LEED v5 Mechanism	Interpretive Result
Integrated governance	BBT 01 K1; BBT 01 K3; BBT 02 K1 (mandatory)	IPc1 Integrative Design Process; PRc2 LEED AP	Shared integrative-process logic; YeS-TR embeds nationally defined project roles.
Energy planning/performance	BBT 02 K2; EKV 01 K1 (minimum energy performance mandatory)	IPp3 Carbon Assessment; EAp1; EAp2	Similar early-analysis/minimum-performance logic; YeS-TR is directly linked to national reference-building/BEP-TR procedures.
Water	BBT 02 K3; SAY 01 K1	WEp1/WEp2; WEc1/WEc2	Similar quantified water-efficiency goals, but baseline/enhanced requirements are packaged differently.
Indoor environmental quality	İOK 01 K1, K4, K5; İOK 03 K1; İOK 04 K1	EQp2; EQc1; EQc2	Both address indoor air quality (IAQ), daylight, visual and thermal comfort; YeS-TR separates several technical requirements that LEED partly aggregates.
Worker/social protection	BBT 04 K3 (mandatory)	EQp1 Construction Management	Partial match: EQp1 has broader construction management/environmental aims; it is not equivalent to the YeS-TR worker H&S requirement.
Site and mobility	BBT 01 K4	LTc1–LTc4; SSp1/SSc1	YeS-TR combines site/transport concerns within a linked criterion; LEED distributes them across dedicated categories.

Note: YeS-TR findings refer only to the 15 audited criteria; no full-system weighting or ranking is inferred. YeS-TR status and implementation evidence are based on the current national framework and 2026 guidance [28,29]. LEED comparators use the February 2026 rating system [30]. The July scorecard and addenda [26,27] were screened for membership, status and maximum points; the HST coding remains a February-edition snapshot.

**Table 5:** Verified milestones in the evolution of green building certification governance in Türkiye, 2020–2026.

Period/Date	Verified Milestone	Analytical Significance
2020 baseline	The earlier study characterized BREEAM and LEED as major reference systems in Türkiye and described domestic certification initiatives as developing/consolidating.	Historical starting point: international systems prominent; national certification not yet a mature regulatory instrument.
2021–2022	YeS-TR implementation procedures and the national Green Certificate framework were operationalized; the 2022 regulation formalized the national system and certification structure.	Institutionalization of a nationally administered certification mechanism.
2 Dec 2025	The Green Certificate Regulation and assessment guides were updated; certification criteria and professional qualification processes were revised.	Current regulatory and criterion framework established for the 2026 analysis.
1 Jan 2026	Green Certificate certification became mandatory for new public buildings with total construction area ≥ 10,000 m^2^.	Transition from voluntary certification toward mandatory application for a defined public-building segment.
2 Mar 2026	The Ministry published the Public Buildings Green Certificate Implementation Guide.	Operational integration of YeS-TR criteria with design and construction stages.
13–16 Mar 2026	A strategic cooperation protocol was announced for YeS-TR and YeS-INT implementation in projects involving the Ministry, Emlak Konut GYO, and the Türkiye Environment Agency.	Evidence of wider institutional embedding and planned international extension of the national framework.

Note: The timeline reports only milestones supported by the verified 2020 baseline and current official institutional/regulatory sources. It describes changes in governance and regulatory status, not causal effects on building performance or certification uptake. Sources: Yılmaz and Balyemez [23]; the Ministry’s official announcements [28,29,34,35,36] and the regulatory amendment [24].

**Table 6:** Sensitivity of LEED v5 HST profiles to primary-only and fractional multidomain allocation.

HST Domain	DCI Primary	DCI Fractional	NWS Primary	NWS Fractional	MRD Primary	MRD Fractional
HST-1	22.4%	20.4%	26.4%	27.3%	31.3%	25.0%
HST-2	12.2%	11.2%	14.5%	12.3%	12.5%	12.5%
HST-3	8.2%	7.1%	8.2%	9.1%	12.5%	9.4%
HST-4	6.1%	7.1%	2.7%	6.4%	6.3%	3.1%
HST-5	12.2%	12.2%	10.9%	10.9%	12.5%	12.5%
HST-6	6.1%	4.1%	10.9%	7.3%	0.0%	0.0%
HST-7	6.1%	6.1%	5.5%	3.2%	0.0%	6.3%
HST-8	10.2%	15.3%	5.5%	5.0%	12.5%	25.0%
HST-9	6.1%	7.1%	3.6%	5.0%	6.3%	3.1%
HST-10	2.0%	2.0%	8.2%	8.2%	0.0%	0.0%
HST-11	8.2%	7.1%	3.6%	5.5%	6.3%	3.1%

Note: Table 6 uses the adjudicated secondary codes for the 0.50/0.50 scenario. Single-domain items retain full weight. The reconciled 1.00/0, 0.75/0.25, 0.50/0.50, and 0.25/0.75 scenarios for both LEED and BREEAM are provided in Supplementary Data S1; original first-round codes and agreement are retained separately.

**Table 7:** Provisional BRL regrouping of primary-domain sustainability coverage in LEED v5 and BREEAM V7.

BRL Dimension	Mapped HST	LEED v5 Primary Coverage (%)	BREEAM V7 Primary Coverage (%)
BRL-1 Resource frugality and circular flows	HST-1, HST-2, HST-3	42.9	47.1
BRL-2 Ecological integration and externalities	HST-4, HST-7	12.2	15.7
BRL-3 Climate adaptation and resilience	HST-9	6.1	5.9
BRL-4 Network and mobility	HST-6	6.1	3.9
BRL-5 Coordination and verification	HST-8	10.2	9.8
BRL-6 Social cooperation and well-being	HST-11	8.2	2.0
Not mapped	HST-5, HST-10	14.3	15.7

Note: LEED and BREEAM percentages use complete criterion/issue denominators. YeS-TR is excluded because the 15 criteria are purposively selected. HST-5 and HST-10 remain unmapped in the provisional BRL.

## Data Availability

Source coding, original and reconciled secondary fields, the codebook, the YeS-TR matched audit, rating-specific minima, HST/BRL scenarios and edition/source screening are in Supplementary Data S1. First-round independent labels, 54 disputed secondary cases, final decisions and reproducible agreement calculations are in Supplementary Data S2. First-round labels remain separate from reconciled sensitivity inputs. Recoding independence and the first-five chronology are confirmed by the corresponding author; the decision files are not an independent timestamped access log.

## Supplementary Materials

The following supporting information can be downloaded at https://www.mdpi.com/article/10.3390/biomimetics11100737/s1. Two supplementary workbooks accompany the manuscript. Supplementary Data S1 (Biomimetics_S1_Coding_and_Sensitivity.xlsx) contains the HST codebook; original and reconciled LEED/BREEAM coding matrices; BREEAM rating-specific minima; purposive YeS-TR matched audit; four-scenario HST and BRL sensitivity calculations; BRL crosswalk; reliability summary; 49-record February-to-July LEED edition screening; and source audit trail. Supplementary Data S2 (Biomimetics_S2_Independent_Recoding.xlsx) contains the 100 original second-coder decisions and rationales, all 54 initial secondary differences, final resolutions, a first-five provenance statement, and formula-based agreement and Cohen’s kappa calculations.

## References

[1] Ilieva L., Ursano I., Traista L., Hoffmann B., Dahy H. (2022). Biomimicry as a Sustainable Design Methodology—Introducing the ‘Biomimicry for Sustainability’ Framework. Biomimetics.

[2] Verbrugghe N., Rubinacci E., Khan A.Z. (2023). Biomimicry in Architecture: A Review of Definitions, Case Studies, and Design Methods. Biomimetics.

[3] Ortega Del Rosario M.D.L.A., Beermann K., Chen Austin M. (2023). Environmentally Responsive Materials for Building Envelopes: A Review on Manufacturing and Biomimicry-Based Approaches. Biomimetics.

[4] Oguntona O.A. (2026). The Push and Pull of Biomimicry in Construction: Identifying Key Drivers for Sustainable Transformation. Biomimetics.

[5] Doan D.T., Ghaffarianhoseini A., Naismith N., Zhang T., Ghaffarianhoseini A., Tookey J. (2017). A critical comparison of green building rating systems. Build. Environ..

[6] Shan M., Hwang B.-G. (2018). Green building rating systems: Global reviews of practices and research efforts. Sustain. Cities Soc..

[7] Awadh O. (2017). Sustainability and green building rating systems: LEED, BREEAM, GSAS and Estidama critical analysis. J. Build. Eng..

[8] Mattoni B., Guattari C., Evangelisti L., Bisegna F., Gori P., Asdrubali F. (2018). Critical review and methodological approach to evaluate the differences among international green building rating tools. Renew. Sustain. Energy Rev..

[9] Suzer O. (2019). Analyzing the compliance and correlation of LEED and BREEAM by conducting a criteria-based comparative analysis and evaluating dual-certified projects. Build. Environ..

[10] Li X., Feng W., Liu X., Yang Y. (2023). A comparative analysis of green building rating systems in China and the United States. Sustain. Cities Soc..

[11] Mohd Ariffin N.A., Gad S.F. (2017). Content Analysis of the Existence of Biomimicry Life’s Principles in Green Building Index Malaysia. Plan. Malays..

[12] Butt A.N. (2022). Biomimicry and the BREEAM Category of Energy for Sustainable Architecture and Sustainable Urbanism. GSC Adv. Res. Rev..

[13] Karabetça A.R. Integration of Biomimicry Design Thinking and the Green Building Certification Systems: LEED as a Case Study. Proceedings of the 4th International Conference on New Trends in Architecture and Interior Design.

[14] Olanrewaju O.I., Enegbuma W.I., Donn M. (2024). Operational, embodied and whole life cycle assessment credits in green building certification systems: Desktop analysis and natural language processing approach. Build. Environ..

[15] Ng W.L., Mulya K.S., Wong K.Y., Tan H., Bao Z., Chen L., Yap P.-S., Jiang P., Woon K.S. (2026). Decarbonization in green building rating systems: A systematic review on embodied and operational carbon credits. J. Environ. Manag..

[16] Arslan V., Ulubeyli S. (2026). Aligning green building rating systems with whole life carbon: A comparative assessment for new buildings. Energy Build..

[17] Kaufmann V., Zhu B. (2026). Beyond environmental metrics: Balancing environmental, social, and governance in building-level sustainability assessment. Clean. Environ. Syst..

[18] De Garrido-Talavera L.A. (2026). CEDES: A taxonomic and LCA-based Green Building Rating System for sustainable building design. Environ. Sustain. Indic..

[19] Dharmarathna D., Graham P., Bunster V. (2025). Unpacking the evolution of Green Star rating tools and their certification trends in Australian building projects. J. Build. Eng..

[20] Tanriverdi Başkir A.N. (2026). Evaluation of a standard residential building in Türkiye in terms of Yes-Tr certification: A credit analysis based on an existing building. Des. Nexus Interdiscip. J. Des. Stud..

[21] Güldağlı B.C. (2026). Sürdürülebilir Kalkınma Hedefleri kapsamında “Yeşil Sertifika Bina” sertifikasyon sisteminin değerlendirilmesi. Recep Tayyip Erdoğan Üniversitesi Fen Mühendislik Bilim. Derg..

[22] Sandikci H.N., Ayas N., Atak Akbulut H., Ülker B., Karacif E. (2026). Yes-INT ve uluslararası yeşil sertifika sistemlerinin gelişmekte olan ülkelerde uygulanabilirliğinin değerlendirmesi. Çevre Şehir İklim Derg..

[23] Yılmaz E., Balyemez S. (2020). Türkiye’de yeşil bina sertifikasyon sistemleri. ABMYO Derg..

[24] Republic of Türkiye Planlı Alanlar İmar Yönetmeliğinde Değişiklik Yapılmasına Dair Yönetmelik. Official Gazette, 11 March 2025, No. 32838, Article 57/A(6); Effective 1 January 2026. https://www.resmigazete.gov.tr/eskiler/2025/03/20250311-1.htm.

[25] Krippendorff K. (2018). Content Analysis: An Introduction to Its Methodology.

[26] U.S. Green Building Council LEED v5 Building Design and Construction Addenda, 31 July 2026.

[27] U.S. Green Building Council (2026). LEED v5 BD+C Rating System.

[28] T.C. Çevre, Şehircilik ve İklim Değişikliği Bakanlığı (2025). “Binalar ile Yerleşmeler İçin Yeşil Sertifikalar Yönetmeliği” Güncellendi. https://meslekihizmetler.csb.gov.tr/haberler/binalar-ile-yerlesmeler-icin-yesil-sertifikalar-yonetmeligi-guncellendi-296755.

[29] T.C. Çevre, Şehircilik ve İklim Değişikliği Bakanlığı (2026). Kamu Binaları İçin Yeşil Sertifika Uygulama Rehberi Yayınlandı. https://meslekihizmetler.csb.gov.tr/haberler/kamu-binalari-icin-yesil-sertifika-uygulama-rehberi-yayinlandi-304019.

[30] U.S. Green Building Council (2026). LEED v5 BD+C Rating System.

[31] BRE Global Ltd (2025). BREEAM New Construction International: Version 7.0.0 Technical Manual (SD6073).

[32] Çevre Dostu Yeşil Binalar Derneği B.E.S.T.: Konut ve Ticari Sertifikası. https://www.cedbik.org/best.

[33] Biomimicry 3.8. Biomimicry DesignLens: Life’s Principles, Generation 6; Biomimicry 3.8: 2013. https://biomimicry.net/the-buzz/resources/designlens-lifes-principles/.

[34] T.C. Çevre, Şehircilik ve İklim Değişikliği Bakanlığı (2021). Binalar ile Yerleşmeler İçin Yeşil Sertifika Uygulama Tebliği Yayımlanmıştır. https://meslekihizmetler.csb.gov.tr/haberler/binalar-ile-yerlesmeler-icin-yesil-sertifika-uygulama-tebligi-yayimlanmistir.

[35] T.C. Çevre, Şehircilik ve İklim Değişikliği Bakanlığı (2022). Binalar ile Yerleşmeler İçin Yeşil Sertifika Yönetmeliği. https://meslekihizmetler.csb.gov.tr/binalar-ile-yerlesmeler-icin-yesil-sertifika-yonetmeligi-haber-272022.

[36] T.C. Çevre, Şehircilik ve İklim Değişikliği Bakanlığı (2026). Bakanlığımızdan YeS-TR ve YeS-INT İçin Stratejik İşbirliği. https://meslekihizmetler.csb.gov.tr/haberler/bakanligimizdan-yes-tr-ve-yes-int-icin-stratejik-isbirligi-304267.

[37] Clay K., Severnini E., Sun X. (2023). Does LEED certification save energy? Evidence from retrofitted federal buildings. J. Environ. Econ. Manag..

[38] Vosoughkhosravi S., Dixon-Grasso L., Jafari A. (2022). The impact of LEED certification on energy performance and occupant satisfaction: A case study of residential college buildings. J. Build. Eng..

